# Combining Intramuscular and Intranasal Immunization With the MF59‐Adjuvanted Respiratory Syncytial Virus Pre‐Fusion Protein Subunit Vaccine Induces Potent Humoral and Cellular Immune Responses in Mice

**DOI:** 10.1002/mco2.70301

**Published:** 2025-07-15

**Authors:** Jie Shi, Hong Lei, Yu Zhang, Chunjun Ye, Xiya Huang, Yishan Lu, Yanyan Liu, Jian Liu, Danyi Ao, Yingqiong Zhou, Jiong Li, Guangwen Lu, Xiangrong Song, Xiawei Wei

**Affiliations:** ^1^ Laboratory of Aging Research and Cancer Drug Target, State Key Laboratory of Biotherapy and Cancer Center, National Clinical Research Center for Geriatrics, West China Hospital, Sichuan University Chengdu Sichuan People's Republic of China

**Keywords:** intranasal immunization, MF59 adjuvant, mucosal immunity, respiratory syncytial virus (RSV), RSV pre‐fusion (preF) protein

## Abstract

Respiratory syncytial virus (RSV) ranks as the second leading cause of infant death globally and a significant contributor to morbidity and mortality among adults over 60 years old. The development of effective RSV vaccines and immunoprophylaxis remains a key focus. In our research, we formulated a protein‐based vaccine known as MF59/preF, which combines the RSV pre‐fusion (preF) antigen with an MF59‐like oil‐in‐water adjuvant. Intramuscular (IM) or intranasal (IN) immunization of the MF59‐adjuvanted preF protein vaccine elicited robust immune responses and neutralizing antibodies against both RSV A2 and RSV B strains, with the IM showing a particularly pronounced effect. Notably, IN immunization with MF59/preF demonstrated superior mucosal immunity, characterized by elevated levels of IgA antibodies and an increased frequency of tissue‐resident memory T (T_RM_) cells locally. More importantly, the combined IM and IN delivery of the MF59/preF vaccine synergistically enhanced antigen‐specific humoral and cellular immune responses at both systemic and mucosal sites. Our study highlights the crucial impact of the route of administration and adjuvanted‐protein subunit vaccines on triggering strong humoral and cellular immunity in mice.

## Graphical abstract

In our study, we formulated a protein‐based vaccine known as MF59/preF, which contains RSV pre‐fusion (preF) protein antigen in combination with an MF59‐like oil‐in‐water adjuvant. Three‐dose intramuscular (IM) administration of this vaccine induced robust humoral and cellular immunity against RSV F protein in systemic immune organs rather than at local sites. Intranasal (IN) immunization with MF59/preF demonstrated superior mucosal immunity, characterized by elevated levels of secretory IgA antibodies and an increased frequency of tissue‐resident memory T (T_RM_) cells locally. We then optimized the MF59/preF vaccine by combining IM and IN immunization, which induced stronger systemic and mucosal immunity.

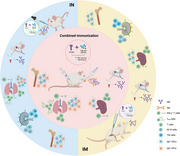

## 1 Introduction

Respiratory syncytial virus (RSV) stands out as a leading culprit behind worldwide acute respiratory illnesses, resulting in over 33 million cases, approximately 3.6 million hospitalizations, and more than 100,000 deaths annually [1, 2]. All age groups remain vulnerable to RSV infection, with particularly high susceptibility observed among infants, pregnant women, the elderly, and the immunocompromised [3]. Nearly all children contract RSV by Age 2, with infants under 6 months accounting for roughly half of hospitalizations and fatalities [4, 5]. Severe manifestations are frequently observed in older adults and people suffering from preexisting health issues such as chronic obstructive pulmonary disease (COPD), diabetes, coronary artery disease, and end‐stage renal disease [6, 7]. Unfortunately, antiviral treatment options for RSV are limited [8]. Thus, advancing safe and efficacious vaccines continues to be a critical public health imperative.

Currently, about 33 RSV vaccines and immunoprophylaxis are in clinical trials, employing diverse strategies such as live‐attenuated vaccines, recombinant protein subunit vaccines, adenovirus vector vaccines, chimeric vaccines, nucleic acid vaccines, and monoclonal antibodies [9]. Among the various approaches, protein subunit vaccines, consisting of purified pathogen fragments without the full genome, inherently provide a non‐virulent and highly safe immunization strategy [10]. Studies have shown that RSV recombinant protein subunit vaccines induce potent neutralizing antibodies, stimulate antigen‐specific cellular immunity, and can protect against RSV challenge [11, 12]. Currently, three RSV vaccines are licensed: Arexvy (GSK) [13], Abrysvo (Pfizer) [14], and mRESVIA (Moderna) [15]. The first two are protein‐based subunit vaccines, underscoring the clinical promise of protein subunit vaccines. Although currently approved protein subunit RSV vaccines have shown solid safety and effectiveness, they are administered intramuscularly and primarily induce systemic immune responses characterized by serum IgG production. However, they have limited capacity to stimulate robust mucosal immunity, particularly in inducing IgA production, which is crucial for blocking RSV entry at the primary site of infection [16]. Considering the pivotal role of mucosal immunity in restricting viral replication and spread, novel vaccine strategies that can trigger robust systemic and mucosal immune responses are urgently required to achieve broader and longer‐lasting protection against RSV.

Moreover, subunit vaccines offer a significant safety advantage, as they consist solely of purified pathogen fragments without any replicating genetic material [17]. However, this characteristic may reduce immunogenicity, requiring adjuvants to boost the immune response [18]. Adjuvants are essential for amplifying and sustaining vaccine‐induced immunity while also minimizing antigen dosage and decreasing the necessary number of immunizations [19]. Currently approved adjuvants for human vaccines include alum (aluminum as a mineral salt), MF59 and AS03 (both oil‐in‐water emulsions), AS04 (alum‐adsorbed TLR4 agonist), and alhydroxiquim‐II (alum with TLR7/8 agonist) [20]. MF59, the first oil‐in‐water emulsion adjuvant approved for human application, demonstrates outstanding safety and proven immunogenicity. Administered via injection, MF59 has been effectively applied in both seasonal and pandemic influenza vaccines [21, 22, 23]. In addition, our previous research has shown that the RBD‐HR/trimer combined with an MF59‐like oil‐in‐water adjuvant elicited strong humoral and cellular responses [24, 25], underscoring the value of MF59‐like adjuvants for next‐generation subunit vaccines.

Additionally, the method of administration greatly affects local and systemic immune activation and overall efficacy. Traditional protein subunit vaccines are mainly delivered intramuscularly, which predominantly induces serum IgG but offers limited mucosal immunity [26, 27]. It has been demonstrated that mucosal immunity effectively stimulates IgA production, thereby protecting against viral invasion and spread [28, 29]. Furthermore, studies emphasize that RSV‐specific IgA antibodies generated in the nasal cavity are essential for preventing infection, while serum antibodies exhibit comparatively lower efficacy [30, 31]. Currently, multiple nasal spray vaccines have already obtained approval for preventing influenza and SARS‐CoV‐2 infections. These vaccines exhibit enhanced mucosal immunity by inducing IgA antibodies and T_RM_ cells [32, 33, 34, 35]. Nonetheless, IN vaccination alone is less effective than IM at inducing strong systemic immunity [36, 37], underscoring the value of strategies that combine both routes. For instance, research shows that combining IM and IN immunizations elicits enhanced systemic and mucosal immune responses [38]. Likewise, sequential IN boosting effectively promotes mucosal IgA production through direct IgG to IgA class switching [39]. These findings highlight the potential of hybrid immunization strategies in achieving a more balanced and comprehensive immune response against respiratory viruses.

The RSV fusion (F) glycoprotein facilitates viral entry into host cells and constitutes a pivotal antigen for vaccine design [40, 41]. The F protein exists in two forms: pre‐fusion (preF) and post‐fusion (postF), with the most effective neutralizing antibodies in human serum targeting the preF form [42]. Furthermore, research confirms that the RSV preF protein elicits stronger RSV‐neutralizing antibody responses than the postF form [43, 44, 45]. In this study, we formulated a protein subunit vaccine, MF59/preF, incorporating the RSV preF antigen with MF59‐like oil‐in‐water adjuvant. Immunization via both IM and IN routes elicited robust neutralizing antibodies in serum. IM administration alone failed to induce mucosal immunity, whereas IN alone generated a limited systemic response. Notably, the combined IM‐IN regimen produced enhanced systemic and mucosal immunity. These results underscore the promise of MF59‐adjuvanted RSV preF as a vaccine candidate and highlight the advantage of multimodal immunization strategies for comprehensive immune protection.

## 2 Results

### 2.1 IM Immunization With MF59/preF Vaccine Induces Systemic Humoral and B‐Cell Immune Responses

**FIGURE 1:**
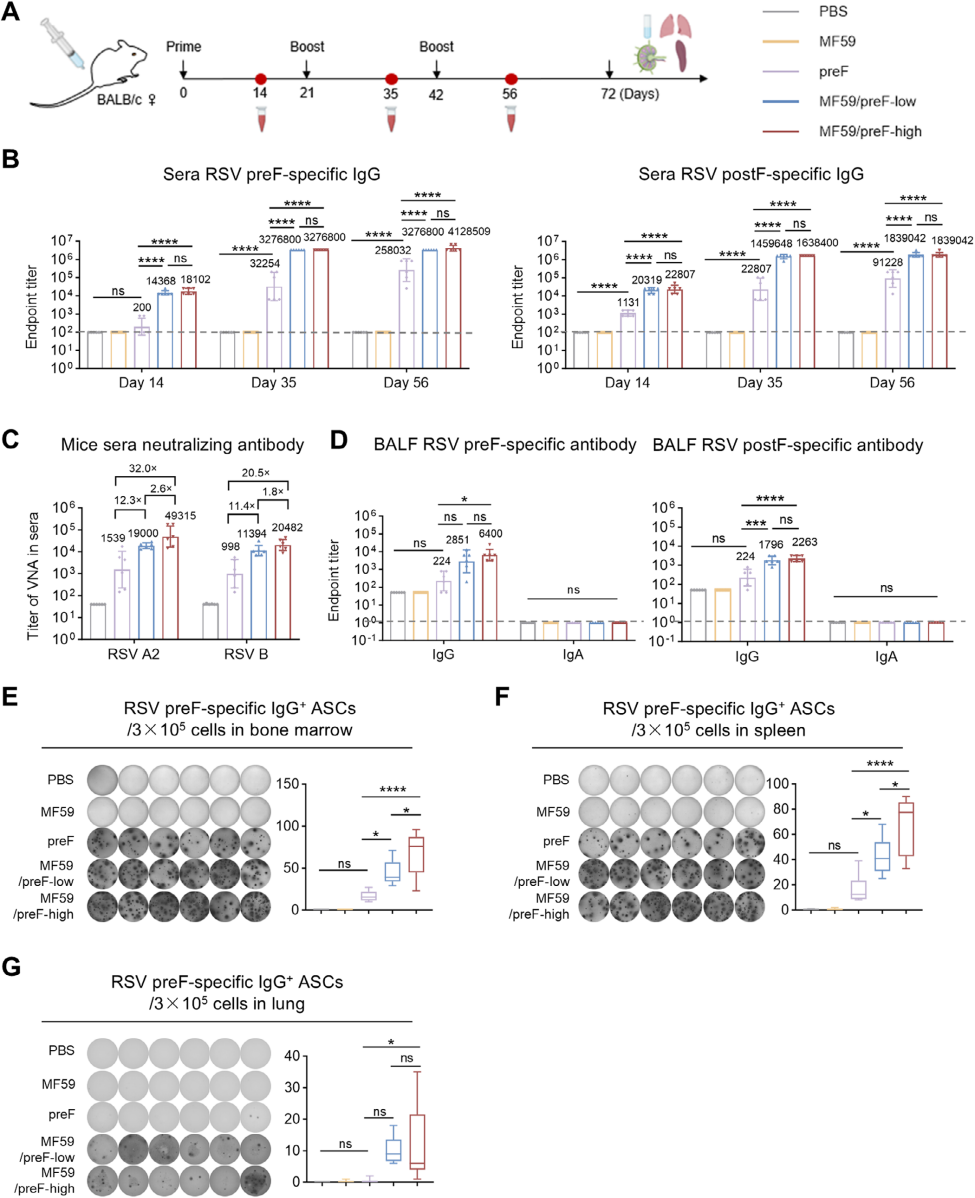
IM immunization with MF59/preF vaccine induced systemic humoral and B‐cell immune responses. (A) The schematic representation of the mouse immunization and sample collection protocol. Mice were immunized intramuscularly with PBS, MF59, preF, MF59/preF‐low, or MF59/preF‐high on Days 0, 21, and 42. Sera were collected on Days 14, 35, and 56, and BALF, spleen, lung, and ILN were harvested on day 72. (B) Endpoint titers of anti‐preF/postF IgG in sera from mice intramuscularly immunized with adjuvanted preF on Days 14, 35, and 56. (C) Titers of virus neutralizing antibody (VNA) against RSV A2 and RSV B in sera collected on Day 56. (D) Endpoint titers of anti‐preF/postF IgG and IgA in BALF collected on Day 72. (E–G) The representative images and quantitative analysis of preF‐specific IgG^+^ ASCs in bone marrow (E), spleen (F), and lung (G). Data are presented as geometric mean values ± SD in B–D. The middle line indicates the median while the whisker shows the data range in E–G. *n* = 6 mice per group. *p* values were conducted by One‐way ANOVA analysis followed by Tukey's multiple comparisons test in B, and D–G. ****p < 0.0001; ***p < 0.001; **p < 0.01; *p < 0.05; ns, not significant.

A robust antibody response has always been a hallmark of an ideal vaccine [46]. Therefore, we first evaluated the antibody response of the MF59/preF vaccine following IM immunization. BALB/c mice in the low‐ and high‐dose groups received IM vaccinations with 5/10 µg of the adjuvanted RSV preF protein vaccine, respectively. The control group was given PBS, MF59, or 10 µg of RSV preF protein alone. The immunization schedule was designed following a prime‐boost regimen, wherein the intervals between administrations were set at 21 days (Figure 1A). An initial injection was carried out on Day 0, and two booster inoculations were subsequently performed on Days 21 and 42, respectively (Figure 1A). Serum was obtained 14 days post each immunization (Days 14, 35, and 56), while BALF was obtained on Day 72 to assess preF‐ and postF‐specific IgG and IgA. ELISA data revealed that IM immunization with preF protein alone induced low preF‐ and postF‐specific IgG levels in Day 56 serum, with IgG titers reaching 10^4^ for postF and 10^5^ for preF, whereas the MF59‐like adjuvanted vaccine significantly augmented the production of IgG antibodies, with titers > 10^6^ (Figure 1B). Furthermore, serum anti‐preF and anti‐postF IgG levels showed no significant differences between the low‐ and high‐dose MF59/preF groups (Figure 1B).

Neutralizing antibodies can effectively neutralize pathogens, diminish pathogen titers, and safeguard tissues or cells from being infected [47]. Subsequently, we conducted live virus neutralization assays on Day 56 serum to evaluate neutralizing capacities triggered by the MF59/preF vaccine. To be more specific, the high‐dose group exhibited GMTs of 49,315 and 20,482 against RSV A2 and RSV B, respectively, while the low‐dose group showed GMTs of 19,000 and 11,394; these differences were not significant (Figure 1C). Compared to preF protein alone, the GMTs were elevated by 32.0‐ and 20.5‐fold in the high‐dose group and by 12.3‐ and 11.4‐fold in the low‐dose group for RSV A2 and RSV B, respectively (Figure 1C). These findings demonstrate that the MF59 adjuvant is essential and that 5 µg of preF protein suffices to generate a robust serum humoral response.

Moreover, local antibody responses, including antigen‐specific IgG and IgA, were assessed. The outcomes manifested that the intramuscularly immunized MF59/preF vaccine was only capable of eliciting IgG antibodies instead of IgA in BALF (Figure 1D), which implied that this vaccine failed to stimulate a localized immune response after IM administration. The detectable IgG antibodies presumably reached the bronchi through the bloodstream.

Antibodies produced by effector B cells upon vaccination are essential for providing protection [48]. These antibodies can neutralize pathogens, enabling other immune cells to recognize and eliminate them. Thus, we verified the activation of the systemic antigen‐specific B cell response following vaccination. Four weeks after the last vaccination, bone marrow, spleen, and lung samples were subjected to ELISpot analysis. The results showed that irrespective of dose, IM administration of MF59/preF induced abundant preF‐specific IgG^+^ antibody‐secreting cells (ASCs) in bone marrow (Figure 1E) and spleen (Figure 1F) but relatively few in the lung (Figure 1G), whereas preF alone generated fewer ASCs in all tissues. Noticeably, IM immunization failed to trigger the generation of preF‐specific IgA^+^ ASCs within any of these tissues (Figure S1A–C). This finding further corroborated that the MF59/preF vaccine, when administered through IM immunization, drives systemic IgG^+^ B cell immunity but is ineffective in stimulating IgA^+^ B cells to synthesize mucosal antibodies.

### 2.2 IM Immunization With MF59/preF Vaccine Induces Systemic T‐Cell Immune Responses

**FIGURE 2:**
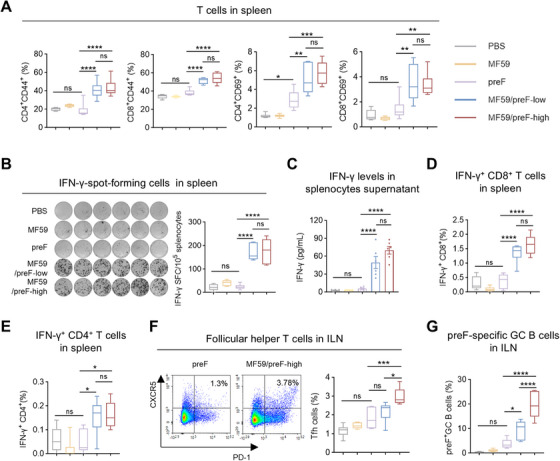
IM immunization with MF59/preF vaccine induced systemic T‐cell immune responses. (A) The percentages of antigen‐experienced (CD44^+^) and activated (CD69^+^) CD4^+^ and CD8^+^ T cells in the spleen. (B) IFN‐γ‐spot‐forming cells in the spleen. Images (left) and quantification (right) of IFN‐γ‐spot‐forming cells were displayed. (C) The levels of IFN‐γ in the supernatants of splenic lymphocytes. (D–E) The percentages of antigen‐specific IFN‐γ producing memory CD8^+^ (D) and CD4^+^ (E) T cells in the spleen. (F–G) The percentages of Tfh cells (CD4^+^CXCR5^+^PD‐1^+^) (F) and preF‐specific GC B cells (B220^+^GL‐7^+^CD95^+^) (G) in ILN. Data are presented as mean values ± SEM in C. The middle line indicates the median while the whisker shows the data range in A–B and D–G. *n* = 6 mice per group. *p* values were conducted by One‐way ANOVA analysis followed by Tukey's multiple comparisons test in A–G. ****p < 0.0001; ***p < 0.001; **p < 0.01; *p < 0.05; ns, not significant.

Vaccination‐induced T‐cell immunity is crucial for combating infections [49]. Effective responses from both CD4^+^ and CD8^+^ T‐cells are essential in preventing pathogen invasions [50]. Consequently, we further investigated T cell activation following IM vaccination. Four weeks after the last vaccination, splenic lymphocytes were isolated and stimulated in vitro with RSV preF protein. Flow cytometry (FCM) analysis revealed that, compared to the preF protein alone, the MF59‐adjuvanted RSV preF formulation markedly increased the proportions of antigen‐experienced (CD44^+^) and activated (CD69^+^) CD4^+^ and CD8^+^ T cells in the spleen, whereas the naked preF induced only a slight rise in CD4^+^ CD69^+^ T cell frequency (Figure 2A). Subsequently, ELISpot further demonstrated that the MF59/preF vaccine induced significantly more IFN‐γ‐producing cells than the naked preF protein (Figure 2B). Moreover, splenic lymphocyte culture supernatants showed significantly elevated IFN‐γ levels in the low/high‐dose group (Figure 2C). Intracellular cytokine staining (ICS) was utilized to further characterize the cell types responsible for IFN‐γ production. We observed increased frequencies of IFN‐γ‐producing CD8^+^ (Figure 2D) and CD4^+^ (Figure 2E) T cells within the spleens of mice administered MF59/preF intramuscularly, whereas preF alone showed no such increase in cytokine‐secreting cells (Figure 2B,D,E) or cytokines (Figure 2C). These findings demonstrate that IM delivery of the MF59/preF vaccine induces robust systemic antigen‐specific T cell activation, unlike the unadjuvanted preF protein.

Germinal centers (GC) are sites of antibody diversification and maturation, generating long‐lasting plasma cells and memory B cells that provide immunity against subsequent infections [51]. Follicular helper T (Tfh) cells are pivotal in initiating GC development and producing antibodies with high affinity and memory B cells [52]. Subsequently, we evaluated the Tfh (CD4^+^CXCR5^+^PD‐1^+^) and preF‐specific GC B (B220^+^GL7^+^CD95^+^) cell responses in ILN. As expected, high‐dose MF59/preF vaccination generated markedly higher levels of both Tfh (Figure 2F) and antigen‐specific GC B cells (Figure 2G) in ILN compared to the preF protein alone group. This augmentation is crucial for the induction of a powerful and enduring humoral immunity against RSV [53]. These findings demonstrated that IM administration of the MF59/preF vaccine can confer an excellent systemic cellular immune reaction.

### 2.3 IN Immunization With MF59/preF Vaccine Induces Local Humoral and B‐Cell Immune Responses

**FIGURE 3:**
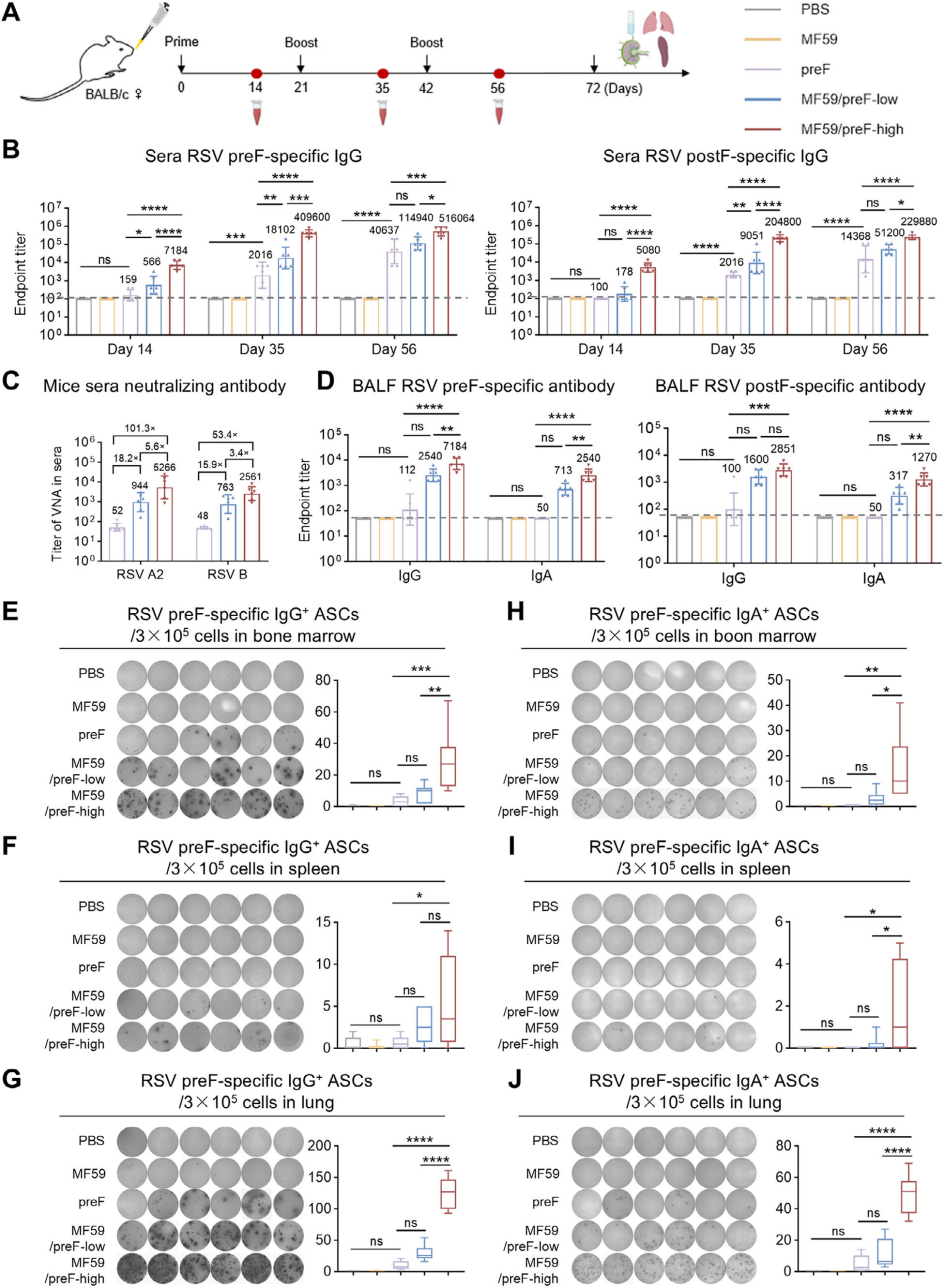
IN immunization with MF59/preF vaccine induced local humoral and B‐cell immune responses. (A) The schematic representation of the mouse immunization and sample collection protocol. Mice were immunized intranasally with PBS, MF59, preF, MF59/preF‐low, or MF59/preF‐high on Days 0, 21, and 42. Sera were collected on Days 14, 35, and 56, and BALF, spleen, lung, and mLN were harvested on Day 72. (B) Endpoint titers of anti‐preF/postF IgG in sera from mice intranasally immunized with adjuvanted preF on Days 14, 35, and 56. (C) Titers of virus neutralizing antibody (VNA) against RSV A2 and RSV B in sera collected on Day 56. (D) Endpoint titers of anti‐preF/postF IgG and IgA in BALF collected on Day 72. (E–J) The representative images and quantitative analysis of preF‐specific IgG^+^ (left) and IgA^+^ (right) ASCs in bone marrow (E, H), spleen (F, I), and lung (G, J). Data are presented as geometric mean values ± SD in B–D. The middle line indicates the median while the whisker shows the data range in E–J. *n* = 6 mice per group. *p* values were conducted by One‐way ANOVA analysis followed by Tukey's multiple comparisons test in B, and D–J. ****p < 0.0001; ***p < 0.001; **p < 0.01; *p < 0.05; ns, not significant.

The MF59 adjuvant, commonly administered intramuscularly, boosts immunity in influenza vaccines [54, 55]. Recent studies indicate that IN administration of MF59 also elicits robust local IgA responses, making it a promising strategy against respiratory viruses by promoting IgA and T_RM_ cells critical for mucosal immunity [56, 57, 58]. Accordingly, we evaluated the immune response elicited by MF59/preF, delivered intranasally, using the same schedule as in the IM study (Figure 3A). We first examined serum IgG production on Days 14, 35, and 56. As depicted in Figure 3B, a single IN immunization with the high‐dose vaccine elicited an elevation in antigen‐specific IgG within the serum (especially preF‐specific), whereas preF protein alone had little effect (Figure 3B). On the 56th day, the serum anti‐preF and anti‐postF IgG titers in the high‐dose group exceeded 10^5^, being > 4‐fold and > 10‐fold greater than those in the low‐dose and naked protein groups, respectively (Figure 3B). Significant differences were observed between the high‐dose group and the remaining two groups (Figure 3B). Additionally, naked preF protein booster immunization elicited modest serum antigen‐specific IgG responses (Figure 3B). Notably, IN delivery of the MF59/preF vaccine‐induced antigen‐specific IgG titers approximately 10‐fold lower than IM immunization (Figure 1B).

Subsequently, we performed the live virus neutralization assays using the serum on Day 56 to evaluate the neutralizing capacities elicited by IN immunization with the MF59/preF vaccine. IN administration of 10 µg RSV preF protein alone merely elicited a negligible neutralization response, with GMTs of 52 and 48 against RSV A2 and RSV B, respectively (Figure 3C). Conversely, the high‐dose MF59/preF vaccine generated GMTs that were 101.3‐ and 53.4‐fold higher than the naked preF group against RSV A2 and RSV B, respectively, and 5.6‐ and 3.4‐fold higher than the low‐dose group (Figure 3C). In addition, unlike IM injection (Figure 1B,C), only the high‐dose MF59/preF vaccine administered intranasally can induce a sufficiently strong serum humoral immune response. Consistent with the IgG antibody, the neutralizing antibodies induced by IN inoculation of the same dose of the MF59/preF vaccine were 10‐fold lower than those by IM inoculation (Figure 1C).

Mucosal antibodies, especially IgA, are vital for frontline defense against localized viral infections [59]. Thus, we further assessed antigen‐specific IgG and IgA in BALF from mice immunized intranasally. In line with serum antibody data, high‐dose MF59/preF vaccination elicited elevated IgG and IgA titers in BALF (Figure 3D), demonstrating that IN administration of MF59/preF induces a stronger local antibody response (particularly IgA) compared to IM injection.

To evaluate how IN vaccination influences B cell activation in immunized mice, we employed the ELISpot assay to examine the generation of antigen‐specific IgG^+^ and IgA^+^ ASCs in bone marrow, spleen, and the lung. As shown in Figure 3E–J, IN administration of naked protein and low‐dose vaccine induced negligible IgG^+^ and IgA^+^ ASCs. High‐dose MF59/preF with IN immunization triggered substantial preF‐specific IgG^+^ ASCs in the lung (Figure 3G) and bone marrow (Figure 3E), with lower levels in the spleen (Figure 3F). Compared to IM immunization (Figure S1A–C), the high‐dose group via intranasal administration elicited preF‐specific IgA^+^ ASCs in bone marrow (Figure 3H), spleen (Figure 3I), and lung (Figure 3J), peaking in the lung (Figure 3J), suggesting that the MF59/preF vaccine administered intranasally provoked local and systemic IgA^+^ ASCs, whereas the quantity of IgG^+^/IgA^+^ ASCs in the spleen was relatively weaker.

### 2.4 IN Immunization With MF59/preF Vaccine Induces Local T‐Cell Immune Responses

**FIGURE 4:**
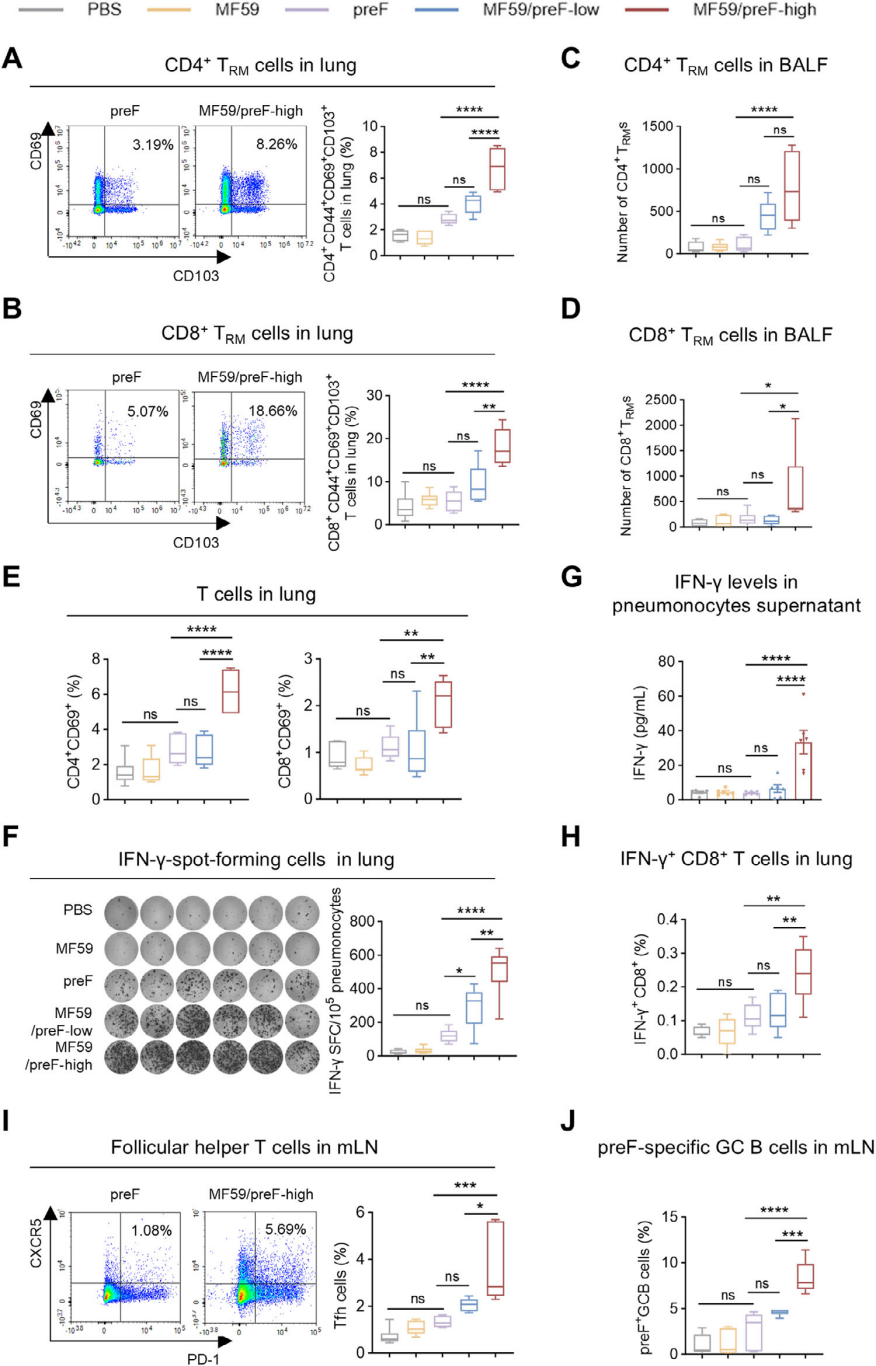
IN immunization with MF59/preF vaccine induced local T‐cell immune responses. (A–D) The proportion of CD4^+^ (A) and CD8^+^ (B) T_RM_ cells in the lung and the absolute number of CD4^+^ (C) and CD8^+^ (D) T_RM_ cells in BALF. T_RM_ cells were gated on CD4^+^CD44^+^CD69^+^CD103^+^ or CD8^+^CD44^+^CD69^+^CD103^+^. (E) The percentages of activated (CD69^+^) CD4^+^ and CD8^+^ T cells in the lung. (F) IFN‐γ‐spot‐forming cells in the lung. Images (left) and quantification (right) of IFN‐γ‐spot‐forming cells were displayed. (G) The levels of IFN‐γ in the supernatants of lung single‐cell suspensions. (H) The percentages of antigen‐specific IFN‐γ‐producing memory CD8^+^ T cells in the lung. (I‐J) The percentages of Tfh cells (CD4^+^CXCR5^+^PD‐1^+^) (I) and preF‐specific GC B cells (B220^+^GL‐7^+^CD95^+^) (J) in mLN. Data are presented as mean values ± SEM in G. The middle line indicates the median while the whisker shows the data range in A– F and H–J. *n* = 6 mice per group. *p* values were conducted by One‐way ANOVA analysis followed by Tukey's multiple comparisons test in A–J. ****p < 0.0001; ***p < 0.001; **p < 0.01; *p < 0.05; ns, not significant.

Apart from IgA antibodies, T_RM_ cells constitute a crucial element of mucosal immunity, furnishing a prompt response to impede viral infection [60, 61]. Therefore, we assessed T_RM_ cell frequencies in BALF and lung tissues of mice receiving IN vaccination. As expected, IN immunization with the high dose of MF59/preF vaccine notably elevated the frequencies of antigen‐experienced CD4^+^/CD8^+^ T_RM_ (characterized by CD44^+^CD69^+^CD103^+^) cells in the lung (Figure 4A,B) and elevated their numbers in BALF (Figure 4C,D). This presented a striking contrast to the naked preF protein and the low‐dose vaccine, neither of which induced the generation of CD4/CD8 T_RM_s.

In the ensuing experiments, we delved deeper into the role of IN immunization with the MF59/preF vaccine in activating T cell responses. Four weeks after the last vaccination, we harvested lung tissues, prepared lung single‐cell suspensions, and then conducted in vitro stimulation using RSV preF protein. FCM analysis indicated that IN administration with the high‐dose of MF59/preF vaccine markedly elevated the percentage of CD4^+^CD69^+^ and CD8^+^CD69^+^ T cells in the lung compared to the naked preF protein and the low‐dose groups (Figure 4E). Furthermore, the high‐dose group showed a significant rise in CD4^+^CD44^+^ T cell proportions relative to the naked protein group, while neither dose affected CD8^+^CD44^+^ T cell levels (Figure S2A). Consistently, the low‐dose vaccine was insufficient to enhance CD4/CD8 expression in the lung (Figure 4E). ELISpot analysis revealed elevated numbers of IFN‐γ‐producing cells in both low‐ and high‐dose MF59/preF groups compared to the naked preF group, with a significant difference observed between the two vaccine doses (Figure 4F). Subsequently, ELISA analysis then detected IFN‐γ in lung cell culture supernatants, finding it present solely in the high‐dose group (Figure 4G). Additionally, ICS analysis revealed comparable outcomes, with the high‐dose group showing enhanced levels of IFN‐γ^+^ CD8^+^ T cells (Figure 4H) but not IFN‐γ^+^ CD4^+^ T cells (Figure S2B). Overall, the data suggest that the low‐dose vaccine elicits only limited IFN‐γ production, whereas robust antigen‐specific T cell induction and activation in the lung occur exclusively with the high‐dose vaccination.

Then, we evaluated the Tfh and preF‐specific GC B cell responses in mLN from intranasally vaccinated mice. As anticipated, IN administration of the high‐dose MF59/preF vaccine markedly elevated Tfh cell (Figure 4I) and preF‐specific GC B cell (Figure 4J) frequencies in mLN, showing significant differences versus both the naked preF and low‐dose groups. These results support the high dose as the preferred option for IN vaccination of the MF59/preF vaccine.

### 2.5 Combination of IM and IN Immunization Using MF59/preF Vaccine Elicits Both Local and Systemic Humoral and B‐Cell Immune Responses

**FIGURE 5:**
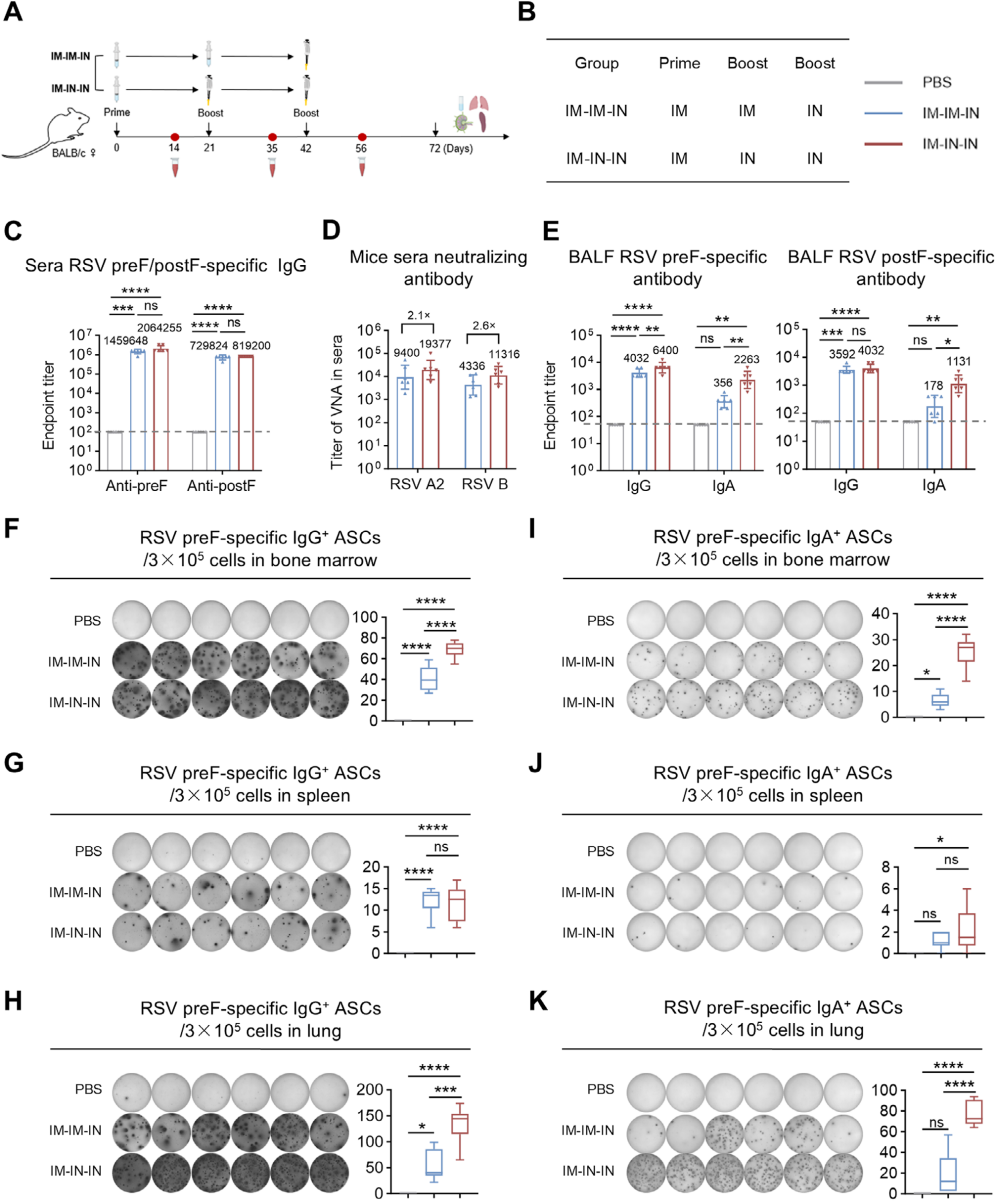
Combination of IM and IN immunization using MF59/preF vaccine elicited both local and systemic humoral and B‐cell immune responses. (A–B) The schematic representation of the mouse immunization and sample collection protocol. BALB/c mice received two intramuscular doses of the MF59/preF vaccine followed by a single intranasal dose (IM‐IM‐IN), or one intramuscular dose followed by two intranasal doses (IM‐IN‐IN). Sera were collected on Days 14, 35, and 56, and BALF, spleen, lung, and mLN were harvested on Day 72. (C) Endpoint titers of anti‐preF/postF IgG in sera on Day 56. (D) Titers of virus neutralizing antibody (VNA) against RSV A2 and RSV B in sera collected on Day 56. (E) Endpoint titers of anti‐preF/postF IgG and IgA in BALF collected on Day 72. (F‐K) The representative images and quantitative analysis of preF‐specific IgG^+^ (left) and IgA^+^ (right) ASCs in bone marrow (F, I), spleen (G, J), and lung (H, K). Data are presented as geometric mean values ± SD in C–E. The middle line indicates the median while the whisker shows the data range in F–K. *n* = 6 mice per group. *p* values were conducted by One‐way ANOVA analysis followed by Tukey's multiple comparisons test in C–K. ****p < 0.0001; ***p < 0.001; **p < 0.01; *p < 0.05; ns, not significant.

The above results indicate that IM vaccination alone does not elicit mucosal IgA, while IN vaccination alone yields markedly lower systemic IgG than the IM route. Therefore, to overcome the limitations of single‐route administration, we implemented a combined IM and IN immunization strategy to enhance the comprehensiveness of the immune response generated by the MF59/preF vaccine. Mice were immunized with a high‐dose prime‐boost strategy at 21‐day intervals (Figure 5A). Mice in the IM‐IM‐IN group were given the initial two doses intramuscularly, followed by an IN booster. In the IM‐IN‐IN group, the primary immunization was IM, and the two booster immunizations were IN (Figure 5B). ELISA results showed that both the IM‐IM‐IN and IM‐IN‐IN regimens elicited robust preF‐ and postF‐specific IgG titers in mouse serum, demonstrating no significant differences between them (Figure 5C).

Live virus neutralization assays were assessed on Day 56 serum to evaluate the neutralizing capacities induced by the MF59/preF vaccine administered via IM‐IM‐IN and IM‐IN‐IN regimens. The IM‐IN‐IN group exhibited GMTs of 19,377 and 11,316 against RSV A2 and RSV B, respectively, representing 2.1‐ and 2.6‐fold increases over the IM‐IM‐IN group (Figure 5D), demonstrating superior neutralizing capacity with the IM‐IN‐IN schedule.

Then, we further assessed antigen‐specific IgG and IgA in BALF following IM‐IM‐IN and IM‐IN‐IN vaccination. Both regimens elevated IgG titers in BALF (Figure 5E), whereas IM‐IN‐IN induced significantly greater IgA levels, indicating that two IN doses are required to achieve robust local IgA responses.

To assess the impact of IM‐IM‐IN and IM‐IN‐IN immunization on local and systemic B cell activation, ELISpot assays measured antigen‐specific IgG^+^ and IgA^+^ ASCs in bone marrow, spleen, and lung. The IM‐IN‐IN regimen significantly induced higher levels of IgG^+^ and IgA^+^ ASCs across all tissues (Figure 5F–K) compared to IM‐IM‐IN, demonstrating that two IN doses following one IM injection elicit stronger B cell responses.

### 2.6 Combination of IM and IN Immunization Using MF59/preF Vaccine Elicits both Local and Systemic T‐Cell Immune Responses

**FIGURE 6:**
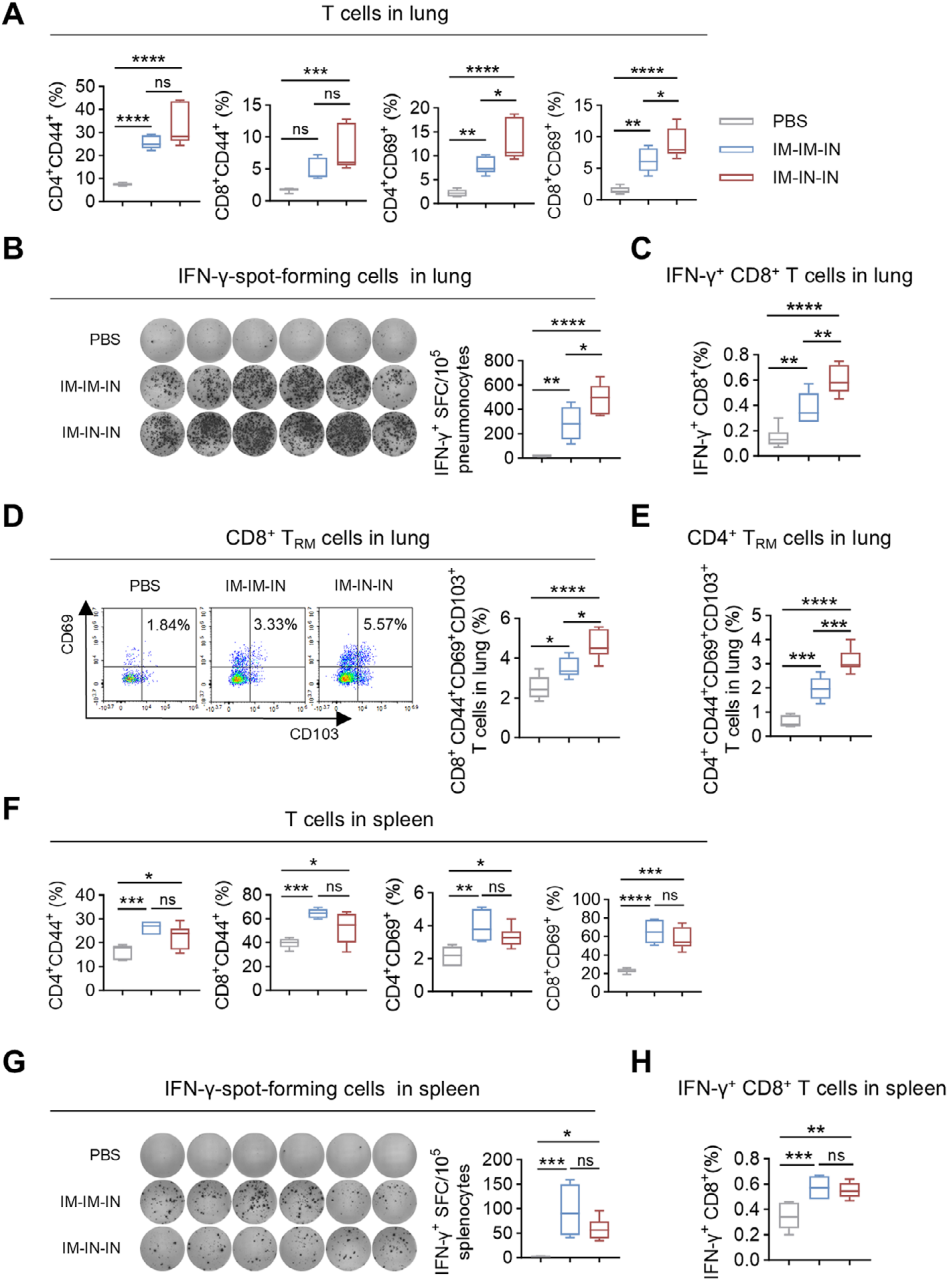
Combination of IM and IN immunization using MF59/preF vaccine elicited both local and systemic T‐cell immune responses. (A) The percentages of antigen‐experienced (CD44^+^) and activated (CD69^+^) CD4^+^ and CD8^+^ T cells in the lung. (B) IFN‐γ‐spot‐forming cells in the lung. Images (left) and quantification (right) of IFN‐γ‐spot‐forming cells were displayed. (C) The percentages of antigen‐specific IFN‐γ‐producing memory CD8^+^ T cells in the lung. (D‐E) The proportion of CD8^+^ (D) and CD4^+^ (E) T_RM_ cells in the lung. T_RM_ cells were gated on CD4^+^CD44^+^CD69^+^CD103^+^ or CD8^+^CD44^+^CD69^+^CD103^+^. (F) The percentages of antigen‐experienced (CD44^+^) and activated (CD69^+^) CD4^+^ and CD8^+^T cells in the spleen. (G) IFN‐γ‐spot‐forming cells in the spleen. Images (left) and quantification (right) of IFN‐γ‐spot‐forming cells were displayed. (H) The percentages of antigen‐specific IFN‐γ‐producing memory CD8^+^ T cells in the spleen. The middle line indicates the median while the whisker shows the data range in A–H. *n* = 6 mice per group. *p* values were conducted by One‐way ANOVA analysis followed by Tukey's multiple comparisons test in A–H. ****p < 0.0001; ***p < 0.001; **p < 0.01; *p < 0.05; ns, not significant.

To evaluate the impact of IM‐IM‐IN and IM‐IN‐IN vaccination schedules on local T cell responses in immunized mice. The IM‐IN‐IN regimen markedly elevated the levels of CD4^+^CD44^+^, CD8^+^CD44^+^, CD4^+^CD69^+^, and CD8^+^CD69^+^ T cells in the lung relative to the PBS control (Figure 6A). Conversely, the IM‐IM‐IN regimen elevated CD4^+^/CD8^+^CD69^+^ and CD4^+^CD44^+^ T cell levels in comparison to the PBS control without affecting CD8^+^CD44^+^ T cells (Figure 6A). Although the overall count of CD4^+^/CD8^+^ CD44^+^ T cells remained comparable, the relative frequency of these T cells in the IM‐IN‐IN regimen was significantly (at least with a tendency) higher than that in the IM‐IM‐IN regimen (Figure 6A). Then, ELISpot results showed more IFN‐γ‐producing cells in the IM‐IN‐IN group, with a statistically significant difference compared to the IM‐IM‐IN group (Figure 6B). The ICS assay showed that the IM‐IN‐IN regimen elicited a higher proportion of pulmonary IFN‐γ^+^CD8^+^ T cells compared to the IM‐IM‐IN regimen (Figure 6C). Similar to the results obtained with three IN immunizations (Figure S2B), neither regimen effectively increased pulmonary IFN‐γ^+^CD4^+^ T cell frequencies (Figure S3A).

Subsequently, we further ascertained the frequencies of T_RM_ cells in the lung following the IM‐IM‐IN and IM‐IN‐IN immunizations. As anticipated, the MF59/preF vaccine delivered by combined IM and IN routes significantly increased antigen‐experienced CD4^+^/CD8^+^ T_RM_ cells in the lung, with the IM‐IN‐IN regimen inducing notably higher levels than IM‐IM‐IN (Figure 6D,E). These results show that the IM‐IN‐IN approach elicits a more robust local T‐cell response.

In the following experiments, we examined how the IM‐IM‐IN and IM‐IN‐IN regimens affected splenic T‐cell responses. Four weeks after the last vaccination, splenic lymphocytes were isolated and stimulated in vitro with RSV preF protein. Both regimens significantly increased the frequencies of CD4^+^CD44^+^, CD8^+^CD44^+^, CD4^+^CD69^+^, and CD8^+^CD69^+^ T cells in the spleen (Figure 6F). Additionally, inoculation with the MF59/preF vaccine via the IM‐IM‐IN and IM‐IN‐IN regimens both resulted in a higher abundance of IFN‐γ‐producing cells in the spleen (Figure 6G) and a greater percentage of splenic IFN‐γ^+^CD8^+^ T cells (Figure 6H), while no increase in splenic IFN‐γ^+^CD4^+^ T cells was detected (Figure S3B). No notable differences were found between the two regimens, indicating that both vaccination strategies effectively triggered strong splenic T‐cell responses.

## 3 Discussion

RSV is a major contributor to global health issues, with over 33 million cases of acute lower respiratory infections occurring each year. This leads to approximately 3.6 million hospitalizations and a tragic death toll of over 100,000 worldwide [1, 2, 62]. The incidence and death rates are significantly elevated in low‐ and middle‐income areas, with RSV‐related child fatalities potentially constituting up to 99% of worldwide deaths [63, 64]. These findings underscore the critical demand for a viable RSV vaccine. Despite 33 RSV prophylactic candidates based on recombinant vectors, subunits, particles, live‐attenuated or chimeric viruses, nucleic acids, and monoclonal antibodies being under clinical evaluation [65], only two preF protein‐based vaccines (Arexvy and Abrysvo) have so far been approved, both delivered intramuscularly [66]. Protein subunit vaccines are favored for their strong safety profiles, but IM administration mainly elicits systemic IgG and rarely induces mucosal immunity. In contrast, IN delivery can trigger robust sIgA responses that block viral entry and limit transmission, making mucosal vaccination an attractive complementary strategy [67]. Previous research has indicated that protection against RSV correlates more with nasal IgA than with serum antibodies, fueling interest in mucosal vaccines [31, 68]. We therefore formulated a protein subunit vaccine, MF59/preF, composed of RSV preF protein adjuvanted with an MF59‐like oil‐in‐water emulsion. We assessed the immune response and effectiveness of a prime‐boost schedule using the MF59/preF vaccine administered through IM, IN, or combined administration pathways. Our data show that the MF59‐like adjuvant significantly enhanced the immunogenicity of RSV preF protein and that combining IM and IN routes elicited the broadest response, including high systemic IgG and neutralizing‐antibody titers, robust mucosal IgA, and increased numbers of T_RM_ cells and effector T cells. Systemic IgG antibodies neutralize circulating virus, while mucosal IgA and T_RM_ cells may inhibit RSV invasion at mucosal sites [16, 69].

IM injection is the most common method to prevent systemic and respiratory infections, mainly inducing systemic immunity with T and B cells [70]. Thus, we initially assessed the immune reactions triggered by IM delivery of the MF59/preF vaccine. Three doses of MF59/preF administered intramuscularly induced a robust systemic immune response (Figures 1 and 2). However, IM immunization with MF59/preF failed to induce IgA in BALF (Figure 1D), preF‐specific IgA^+^ ASCs in any tissue (Figure S1A–C), and antigen‐specific T cell activation in the lung (Figure S1D). Thus, IM immunization with MF59/preF reliably induces systemic but not mucosal immunity, underscoring the need for alternative or combined routes to elicit IgA‐mediated protection at the respiratory mucosa.

Compared with IM vaccination, IN delivery generates robust respiratory mucosal immunity, producing IgA and T_RM_ cells that prevent viral entry and contain transmission [71, 72]. Several influenza and SARS‐CoV‐2 nasal‐spray vaccines have already been licensed [32, 33, 34, 35], but no mucosal RSV vaccine is available. Moreover, the advancement of mucosal vaccines faces significant challenges due to a shortage of safe, potent adjuvants [73, 74]. MF59, the first oil‐in‐water emulsion adjuvant for human use, demonstrates high safety and effectiveness, with IN delivery enhancing both mucosal and systemic immune responses [56, 58, 75]. Thus, we assessed the effectiveness of IN immunization with the MF59/preF vaccine. IN immunization with MF59/preF triggered a decent systemic humoral immune and a robust mucosal immune response (Figures 3, 4), including CD4^+^/CD8^+^ T_RM_ cells in the lung and BALF, along with antigen‐specific T cell activation in the lung. Evidence indicates that establishing T_RM_ within pulmonary tissues and airways significantly accelerates the clearance of RSV from the lungs upon reinfection [76, 77]. Following antigen re‐exposure, T_RM_ cells rapidly release pro‐inflammatory cytokines and transform into effector T cells, boosting local antiviral responses [59]. This is markedly different from the outcome of IM immunization, and IN immunization is instrumental in curbing RSV replication and halting viral dissemination at mucosal entry sites.

Although IM injection is the common route for administering systemic vaccines, its limited capacity to induce robust mucosal immunity restricts its effectiveness in preventing RSV infection and nasal shedding. In contrast, IN delivery activates IgA‐ and T_RM_‐mediated protection in the airways. However, the unique physiological characteristics of the nasal cavity may reduce antigen absorption, potentially limiting vaccine efficacy [78]. As previously described, we observed that IN administration of MF59/preF was suboptimal in eliciting robust systemic cellular immune responses (Figure S2C), which play a pivotal role in effective viral elimination. Previous research indicates that combining IM and IN vaccination could address this limitation [38, 79]. Consequently, we evaluated whether combining IN and IM vaccination improves the immunogenic response of the MF59/preF vaccine. Comparative evaluation of various combination protocols revealed that an initial IM dose followed by two IN boosters with MF59/preF elicited the most robust preF‐specific systemic and mucosal immunity (Figures 5 and 6). This combined regimen delivers strong systemic protection while reinforcing mucosal immunity at the entry site, providing dual defense against RSV. By simultaneously targeting systemic and mucosal compartments, the IM‐IN‐IN schedule stands out as the most effective approach and a promising blueprint for vaccines against other respiratory threats, such as the influenza virus and SARS‐CoV‐2.

However, our research faces certain limitations. In this research, we did not conduct challenge experiments, and thus, the in vivo protective efficacy of the MF59/preF vaccine remains unverified. Additionally, although our study primarily focused on IN and IM delivery, exploring additional routes such as subcutaneous, intradermal, and intravenous administration is essential. Such investigations could offer critical insights for designing future protein subunit vaccines and optimizing immunization regimens.

In conclusion, our findings align with previous reports highlighting the immunological advantages of hybrid IM‐IN immunization strategies, emphasizing the potential of mucosal delivery in enhancing local immune protection. Our study further highlights that the MF59‐like oil‐in‐water adjuvant can markedly boost the immunogenicity of the RSV preF protein and the potential of combining IM priming and IN boosting with the MF59/preF vaccine. This IM‐IN hybrid immunization strategy exhibits a synergistic effect in eliciting both systemic and mucosal immune responses, supporting its potential as an optimal immunization strategy against RSV and other respiratory viruses.

## 4 Materials and Methods

### 4.1 Animals

Specific pathogen‐free (SPF) female BALB/c mice (6–8 weeks old; Beijing Vital River, China) were kept at 22°C–23°C with 45%–55% humidity under a 12 h light/dark cycle and allowed free access to food and water. After at least one week of acclimation, mice were randomly assigned to experimental groups. All procedures were approved by the Sichuan University Animal Care and Use Committee (Chengdu, China).

### 4.2 Vaccination of Mice

MF59‐like adjuvant was formulated as previously described [80]. The BALB/c mice (6 per group) were intramuscularly or intranasally immunized with PBS, MF59‐like adjuvant, preF alone (10 µg, Sino Biological, 11049‐VNAS), MF59/preF‐low (5 µg preF), or MF59/preF‐high (10 µg preF) on Days 0, 21, and 42. Each mouse received 100 µL for IM immunization and 50 µL for IN immunization.

To delve into the synergistic immune impact of a combination of IM and IN vaccines, BALB/c mice were administered three high doses of MF59/preF (10 µg preF) on days 0, 21, and 42. The IM‐IM‐IN group received two IM injections followed by an IN boost, while the IM‐IN‐IN group was primed IM and boosted twice intranasally.

Serum was collected 14 days after each immunization. Four weeks after the last vaccination, BALF, lung, spleen, ILN, mLN, and bone marrow were collected for immune analyses.

### 4.3 Measurement of Antibody by ELISA

Briefly, 96‐well ELISA plates (NUNC‐MaxiSorp, Thermo Fisher Scientific) were coated overnight at 4°C with either 1 µg/mL of RSV preF or postF protein (Sino Biological, 11049‐V49H5‐B). Following this, the plates were washed three times with PBST (PBS containing 0.1% Tween‐20) and then blocked with 1% bovine serum albumin (BSA) at 37°C for 1 h. Serum or BALF samples were serially diluted, added to the wells, and incubated at 37°C for 1 h. After washing, 100 µL/well of horseradish peroxidase (HRP)‐conjugated goat anti‐mouse IgG (Thermo Fisher Scientific, 31430) or IgA (Abcam, ab97235) was added and incubated for another hour at 37°C. Following five washes, 100 µL/well of 3,3′,5,5′‐tetramethylbenzidine (TMB, Thermo Fisher Scientific, 34029) substrate was added and developed for 10 min in the dark. The reaction was stopped with TMB stop solution (Beyotime, P0215), and absorbance was measured at 450 nm with a 630 nm reference using a microplate reader (Spectramax ABS, Molecular Devices) with SoftMax Pro 7.1 software.

### 4.4 ELISpot Assay

The lungs from immunized mice were harvested, minced with ophthalmic scissors, and digested for 1 h at 37°C in RPMI 1640 medium containing 1% Type I (1 mg/mL) and 1% Type IV (0.5 mg/mL) collagenase (Gibco). The digested tissue was passed through 70 µm cell strainers (Corning, USA) and subjected to red blood cell lysis. After two washes with PBS, the resulting cell suspension was resuspended in RPMI 1640 complete medium containing 10% fetal bovine serum (FBS), 1 mM pyruvate, 100 µg/mL streptomycin, 100 U/mL penicillin (all from Gibco), 20 U/mL IL‐2, and 50 µM β‐mercaptoethanol (all from Sigma).

The spleens were homogenized with a syringe piston and passed through 70 µm cell strainers to obtain single‐cell suspensions. Bone marrow cells were flushed from the tibiae and femora with RPMI 1640 medium. Both spleen and bone marrow suspensions were then isolated with a mouse lymphocyte separation medium (Dakewe, China), followed by red blood cell lysis treatment. After two washes with PBS, the isolated lymphocytes were finally resuspended in RPMI 1640 complete medium.

IgG^+^ and IgA^+^ ASCs in bone marrow, spleen, and lung were quantified by ELISpot. Sterile 96‐well plates (Mabtech, Sweden) were first activated with a brief 1 min exposure to 35% ethanol, then washed with PBS. The plates were subsequently coated overnight at 4°C with 3 µg/mL of RSV preF protein. Following a wash step, the plates were blocked with RPMI 1640 complete medium and incubated for 2 h at 37°C. Single‐cell suspensions from the spleen, bone marrow, and lung (3 × 10^5^ cells/well) were added and allowed to incubate overnight at 37°C. The next day, the plates were washed and then incubated with HRP‐conjugated goat anti‐mouse IgG (1:5000) or IgA (1:2000) for 2 h at room temperature. After another wash, the spots were developed using TMB substrate, visualized, and then rinsed to halt the reaction. Finally, the number of spots was recorded with the S6 Ultra M2 ELISpot reader (CTL, USA).

Mouse IFN‐γ‐spot‐forming cells in the lung and spleen were detected following the manufacturer's protocol. Sterile 96‐well ELISpot plates (Mabtech) were washed five times with PBS. Next, the plates were incubated with RPMI 1640 complete medium for 2 h at 37°C. Splenic lymphocytes and single‐cell suspensions from the lungs (1×10^5^ cells/well) were then added to the plates along with RSV preF protein and left overnight at 37°C. After washing with PBS, the plates were treated with detection antibody (R4‐6A2‐biotin) for 2 h at room temperature. Following a second wash, streptavidin‐alkaline phosphatase (ALP, 1:1000) was added and incubated for 1 h at room temperature. Spots were visualized by adding BCIP/NBT‐plus substrate until they appeared, and then the plates were rinsed with water to halt the reaction. The resultant spots were counted using an S6 Ultra M2 ELISpot reader (CTL, USA).

### 4.5 Measurement of IFN‐γ by ELISA

IFN‐γ levels in culture supernatants of splenic lymphocytes and single‐cell suspensions from the lungs were quantified with a commercial ELISA kit (Thermo Fisher Scientific, 88–7314). Briefly, 96‐well plates were coated overnight at 4°C with capture antibody. After washing, the plates were blocked with ELISA diluent for 1 h at room temperature. Next, each sample was added and incubated for 2 h. Following another wash step, the plates were treated with detection antibody for 1 h. Subsequent washes preceded the addition of streptavidin‐HRP, which was left to incubate for 30 min. The reaction was developed using TMB substrate for 15 min before being stopped. Absorbance readings were taken at 450 nm using a microplate reader (Spectramax ABS, Molecular Devices) with SoftMax Pro 7.1 software.

### 4.6 Live RSV Virus Neutralization Assay

The live RSV virus neutralization assay was performed as described previously [81]. Briefly, A549 cells (5 × 10^5^ cells/mL) were seeded in 96‐well plates and cultured for 24 h. Serum samples were heat‐inactivated at 56°C for 30 min and then subjected to 3‐fold serial dilutions in DMEM containing 2% heat‐inactivated FBS. An equal volume of RSV (10^4^ TCID_50_/mL) was introduced to the samples and incubated at 37°C for 1 h. These mixtures were then transferred onto the A549 cell monolayers and incubated for 24 h at 37°C. Post‐incubation, the cells were fixed using a 1:1 acetone/methanol solution at −20°C for 20 min and blocked with PBS containing 1% BSA and 0.1% Tween‐20 for 1 h at 37°C. The next step involved staining with an anti‐RSV F antibody (CHEMSTAN, CSD00024) for 1 h at 37°C. Following PBS washes, an HRP‐conjugated secondary antibody (Abcam, ab6759) was applied for another hour at 37°C. To visualize the results, plates were treated with TrueBlue peroxidase substrate (KPL, 95059–168) for 5 to 15 min at room temperature. The reaction was then halted with PBS, and the resulting spots were counted using an ELISpot Reader (AID, ELR08IFL).

### 4.7 Flow Cytometry

Four weeks after the last vaccination, BALF and lung tissue were harvested to assess the T_RM_ cells. Cells were stained with the following antibodies: PerCP/Cyanine5.5‐CD3 (BioLegend, 100218), BV421‐CD4 (BioLegend, 100412), FITC‐CD8a (BioLegend, 100706), BV510‐CD44 (BioLegend, 103044), PE‐CD69 (BioLegend, 164204), and APC‐CD103 (BioLegend, 121414).

Four weeks after the last vaccination, ILN and mLN were collected to analyze Tfh cells and preF‐specific GC B cells. For Tfh cells in ILN, the cells were stained with PerCP/Cyanine5.5‐CD3, PE‐CD19 (BioLegend, 115508), APC‐CD4 (BioLegend, 100412), FITC‐CD185 (CXCR5) (BioLegend, 145520), and BV421‐CD279 (PD‐1) (BioLegend, 109121). For Tfh cells in mLN, the cells were stained with PerCP/Cyanine5.5‐CD3, PE‐CD4 (BioLegend, 100408), FITC‐CD19 (BD Pharmingen, 553785), APC‐CD185 (CXCR5) (BioLegend, 145506), and BV421‐CD279 (PD‐1). To analyze the prevalence of preF‐specific GC B cells within the ILN and mLN, the cells were first incubated with biotin‐tagged preF protein (Sino Biological, HP17OC1701), followed by staining with PerCP/Cyanine5.5‐CD3, PE/Cyanine7‐B220 (BioLegend, 103222), FITC‐CD95 (BioLegend, 152606), APC‐GL‐7 (BioLegend, 144618), and PE‐anti‐biotin antibodies (BioLegend, 409004).

For ICS, splenic lymphocytes and lung single‐cell suspensions were stimulated with 5 µg/mL RSV preF protein for 12 h. Before cell collection, Brefeldin A (BFA, BD Biosciences) was introduced to inhibit cytokine secretion. Cells were stained with the following antibodies: PerCP/Cyanine5.5‐CD3, APC‐CD4, FITC‐CD8a, PE‐CD69, and BV510‐CD44 for 30 min at 4°C. After fixation and permeabilization (Biosciences, 554715), cells were stained with PE/Cyanine7‐IFN‐γ (BioLegend, 505826) antibody for 1 h at room temperature.

### 4.8 Statistical Analysis

All statistical analyses used GraphPad software Prism 10.1.2, and FCM data analysis was performed with NovoExpress 1.4.1 software (ACEA Biosciences, Inc.). The difference between groups was compared using one‐way ANOVA followed by Tukey's multiple comparisons test. ****p < 0.0001; ***p < 0.001; **p < 0.01; *p < 0.05; ns, not significant.

## Author Contributions

X.W. and X.S. conceived and supervised the research and designed the experiments. G.L. and J.L. prepared the MF59‐adjuvanted preF vaccine. J.S., H.L., Y.Z., and C.Y. prepared the vaccine formulations, carried out animal immunizations, and conducted binding antibody assays. J.S., H.L., Y.Z., C.Y., X.H., Y.L., Y.L., J.L., D.A., and Y.Z. collected serum and BALF samples and performed flow cytometry to assess T_RM_, GC B, and Tfh cell frequencies, as well as T cell immune responses in lung and spleen tissues. X.W., J.S., H.L., C.Y., X.H., Y.L., Y.L., J.L., D.A., and YZ. contributed to data analysis and interpretation and guided the refinement of the study direction and mechanistic insights. X.W., J.S., and H.L. wrote the manuscript. All authors have read and approved the article.

## Ethics Statement

All animal experiments in this study were approved by the Institutional Animal Care and Use Committee of Sichuan University (ethical approval number: 20230227017).

## Conflicts of Interest

The authors declare no conflicts of interest.

## Supporting information




**Supporting File**: mco270301‐sup‐0001‐SuppMat.docx

## Data Availability

The data in this study are available from the corresponding author upon reasonable request.
