# Supplementary material for: Combining Intramuscular and Intranasal Immunization With the MF59‐Adjuvanted Respiratory Syncytial Virus Pre‐Fusion Protein Subunit Vaccine Induces Potent Humoral and Cellular Immune Responses in Mice
Source: MedComm (2020). 2025 Jul 15;6(8):e70301. doi: 10.1002/mco2.70301 (PMC12264085; doi:10.1002/mco2.70301)
Supplement: Supplementary file 1 — Supporting File: mco270301‐sup‐0001‐SuppMat.docx [file MCO2-6-e70301-s001.docx]

Supplementary Information for

# Combining intramuscular and intranasal immunization with the MF59-adjuvanted respiratory syncytial virus pre-fusion protein subunit vaccine induces potent humoral and cellular immune responses in mice

Correspondence author. Xiawei Wei ([xiaweiwei@scu.edu.cn](mailto:xiaweiwei@scu.edu.cn)), and Xiangrong Song ([songxr@scu.edu.cn](mailto:songxr@scu.edu.cn) ).

**Includes:**

Supplemental Figure 1-3

**Supplemental Figures**


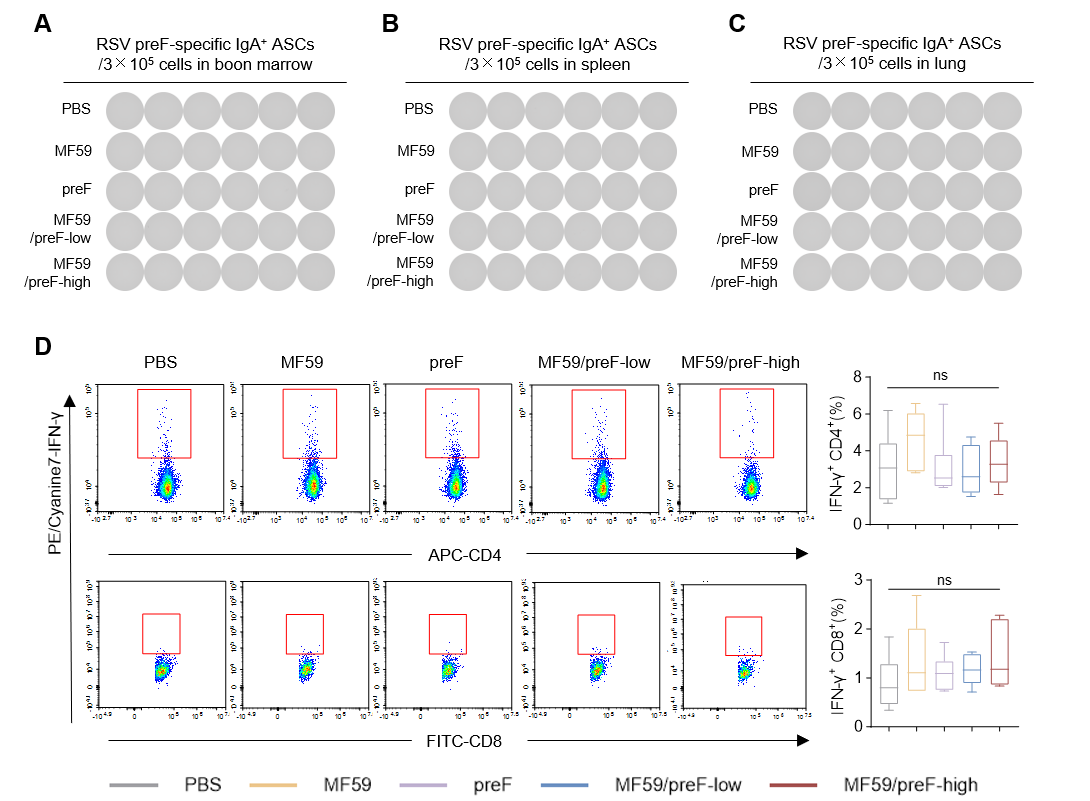


SUPPLEMENTARY FIGURE 1 IM immunization with MF59/preF vaccine failed to induce IgA^+^ B cells and pulmonary T-cell immune responses. (A-C) The representative images and quantitative analysis of preF-specific IgA^+^ ASCs in bone marrow (A), spleen (B), and lung (C). (D) The percentages of antigen-specific IFN-γ producing memory CD4^+^ (up) and CD8^+^ (down) T cells in the lung. The middle line indicates the median while the whisker shows the data range in D. n = 6 mice per group. *P* values were conducted by One-way ANOVA analysis followed by Tukey’s multiple comparisons test in D. ****P* < 0.001; ***P* < 0.01; **P* < 0.05; ns, not signiﬁcant

**
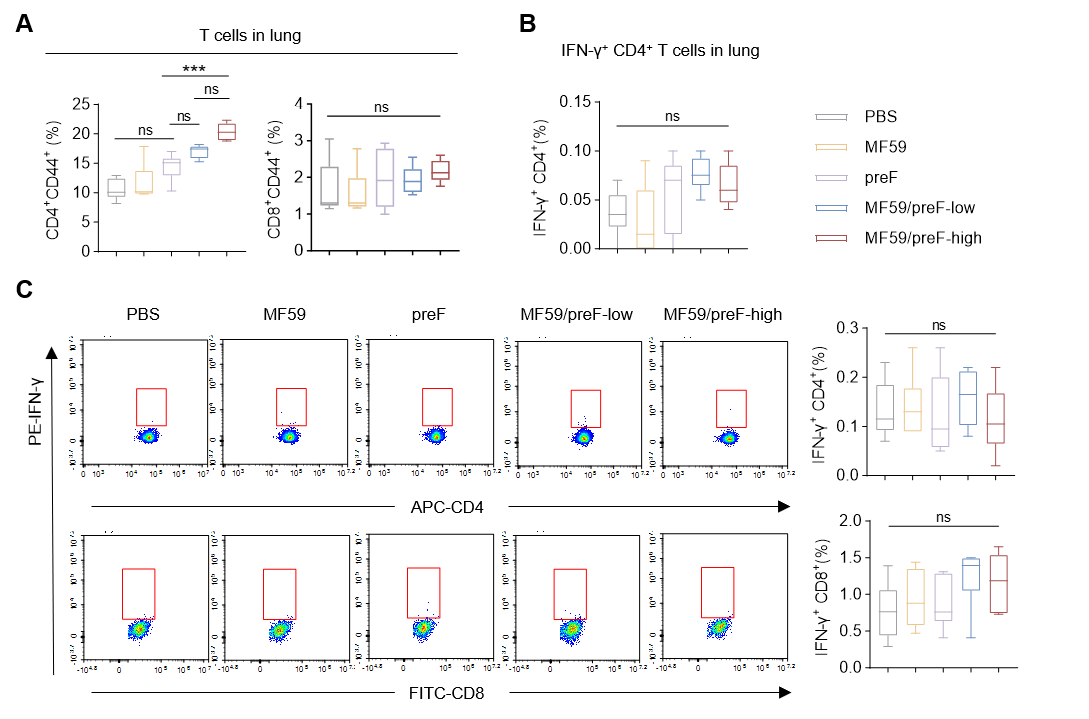
**

SUPPLEMENTARY FIGURE 2 IN immunization with MF59/preF vaccine failed to induce pulmonary antigen-experienced (CD44^+^) T cells, pulmonary antigen-specific IFN-γ producing memory CD4^+^ T cells, and splenic T-cell immune responses. (A) The percentages of antigen-experienced (CD44^+^) CD4^+^ and CD8^+^ T cells in the lung. (B) The percentages of antigen-specific IFN-γ producing memory CD4^+^ T cells in the lung. (C) The percentages of antigen-specific IFN-γ producing memory CD4^+^ (up) and CD8^+^ (down) T cells in the spleen. The middle line indicates the median while the whisker shows the data range in A-C. n = 6 mice per group. *P* values were conducted by One-way ANOVA analysis followed by Tukey’s multiple comparisons test in A-C. ****P* < 0.001; ***P* < 0.01; **P* < 0.05; ns, not signiﬁcant


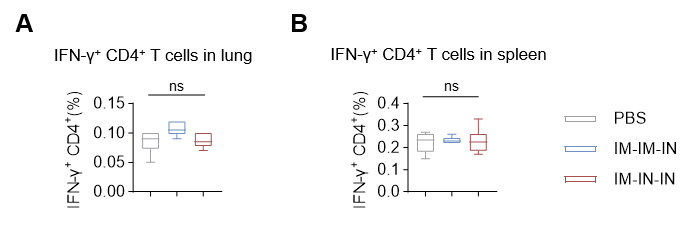


SUPPLEMENTARY FIGURE 3 Combination of IM and IN immunization using MF59/preF vaccine failed to induce antigen-specific IFN-γ producing memory CD4^+^ T cells in the lung and spleen. (A) The percentages of antigen-specific IFN-γ producing memory CD4^+^ T cells in the lung. (B) The percentages of antigen-specific IFN-γ producing memory CD4^+^ T cells in the spleen. The middle line indicates the median while the whisker shows the data range in A-B. n = 6 mice per group. *P* values were conducted by One-way ANOVA analysis followed by Tukey’s multiple comparisons test in A-B. ****P* < 0.001; ***P* < 0.01; **P* < 0.05; ns, not signiﬁcant
